# Endotoxin-induced alterations of adipose tissue function: a pathway to bovine metabolic stress

**DOI:** 10.1186/s40104-024-01013-8

**Published:** 2024-04-06

**Authors:** Miguel Chirivi, G. Andres Contreras

**Affiliations:** Department of Large Animal Clinical Sciences, Michigan State University, East Lansing, MI USA

**Keywords:** Adipose tissue dysfunction, Endotoxin, Inflammation, Insulin resistance

## Abstract

During the periparturient period, dairy cows exhibit negative energy balance due to limited appetite and increased energy requirements for lactogenesis. The delicate equilibrium between energy availability and expenditure puts cows in a state of metabolic stress characterized by excessive lipolysis in white adipose tissues (AT), increased production of reactive oxygen species, and immune cell dysfunction. Metabolic stress, especially in AT, increases the risk for metabolic and inflammatory diseases. Around parturition, cows are also susceptible to endotoxemia. Bacterial-derived toxins cause endotoxemia by promoting inflammatory processes and immune cell infiltration in different organs and systems while impacting metabolic function by altering lipolysis, mitochondrial activity, and insulin sensitivity. In dairy cows, endotoxins enter the bloodstream after overcoming the defense mechanisms of the epithelial barriers, particularly during common periparturient conditions such as mastitis, metritis, and pneumonia, or after abrupt changes in the gut microbiome. In the bovine AT, endotoxins induce a pro-inflammatory response and stimulate lipolysis in AT, leading to the release of free fatty acids into the bloodstream. When excessive and protracted, endotoxin-induced lipolysis can impair adipocyte’s insulin signaling pathways and lipid synthesis. Endotoxin exposure can also induce oxidative stress in AT through the production of reactive oxygen species by inflammatory cells and other cellular components. This review provides insights into endotoxins’ impact on AT function, highlighting the gaps in our knowledge of the mechanisms underlying AT dysfunction, its connection with periparturient cows’ disease risk, and the need to develop effective interventions to prevent and treat endotoxemia-related inflammatory conditions in dairy cattle.

## Introduction

Dairy cows are particularly susceptible to metabolic and inflammatory diseases during the three weeks before and three weeks after parturition. This period, also referred to as the periparturient period, is marked by reduced appetite, heightened energy requirements due to the onset of lactogenesis, and endocrine changes associated with parturition and the initiation of lactation. Limited energy availability results in negative energy balance (NEB), and when excessive, NEB induces the development of metabolic stress. This condition is characterized by enhanced lipolysis in white adipose tissue (AT), increased production of reactive oxygen species (ROS) with limited antioxidant responses, and dysregulated inflammatory responses [1].

The AT is at the center of metabolic stress as this multisite organ stores energy, acts as a reservoir of immune cells, and is an important producer of ROS. The AT is also an endocrine organ that regulates biological functions, including immune function, angiogenesis, glucose homeostasis, food intake, blood pressure, and reproduction [2]. In dairy cattle, as in other mammals, AT regulates systemic metabolism. Any disruption in its functionality increases the risk for metabolic alterations, including insulin resistance (IR), ketoacidosis, and fatty liver, which predispose dairy cows to health problems such as displaced abomasum and retained placenta, and poor milk production and reproductive performance [3, 4].

During the periparturient period AT lipolysis supports part of the energetic cost of milk production [5]. Lipolytic processes induce inflammatory responses within AT characterized by immune cell infiltration, release of lipolytic products, and generation of ROS and other free radicals [6–10]. Similarly, in late lactation cows, feed restriction-induced lipolysis results in enhanced immune cell infiltration within the AT [11]. This adaptive response enhances the production of lipogenic and antioxidant substances that maintain AT homeostasis [2]. However, when adipose tissue becomes insensitive to lipogenic or antioxidative signals, excessive lipolysis and dysregulated inflammation ensue [12]. This heightened inflammatory status predisposes cows to metabolic and infectious diseases [13, 14]. While inflammation plays a significant role in AT dysfunction, the specific factors that trigger the inflammatory process in this organ remain poorly characterized.

Among common causes of inflammatory responses in postpartum dairy cows, endotoxins are likely present during clinical conditions such as mastitis, endometritis, and leaky gut, and if these reach the blood circulation, endotoxemia develops [15]. In humans and rodents, multiple lines of evidence suggest that endotoxemia affects AT function by inducing inflammation, lipolysis dysregulation, oxidative stress, IR, and by altering adipokine secretion [16, 17]. Collectively, these changes may lead to a low-grade chronic inflammation, (also known as “meta-inflammation”), which is associated with an increased risk for disease [18]. This review provides an overview of the current knowledge regarding these changes in white AT of dairy cattle and the possible implications for metabolic and inflammatory functions during endotoxemia in dairy cows. Herein, we describe how endotoxin-induced inflammatory responses may directly modulate AT’s lipolysis and lipogenesis, cytokine production, oxidative stress, and contribute to the development of IR and AT dysfunction. The terms endotoxin and lipopolysaccharide (LPS) are considered synonymous for this review and thus will be used interchangeably.

## Endotoxemia

The term endotoxemia refers to the presence of endotoxins in blood, which is often associated with bacteremia and septicemia [15]. Endotoxin, or LPS, is the major glycolipid found in the outer membrane of Gram-negative bacteria [19]. Endotoxin is a heat-stable molecule containing a pathogen-associated molecular pattern, lipid A. Upon binding to its cell membrane-bound pattern recognition receptor, Toll-like receptor 4 (TLR4), endotoxin triggers a complex signaling cascade, resulting in local or systemic activation of pro-inflammatory mediators and oxidative stress [20, 21]. It is important to note that other bacterial components, such as peptidoglycan (PGN) and lipoteichoic acid (LTA) from Gram-positive bacteria, can also trigger immune activation.

Structural bacterial components can be detected by receptors other than TLR4. The D-glutamyl-meso-diaminopimelic acid (meso-DAP) found in Gram-negative bacteria’s PGN is detected by the cytosolic pattern recognition receptor nucleotide oligomerization domain 1 (NOD1). Similarly, muramyl dipeptide, ubiquitous in the peptidoglycan of Gram-positive bacteria, is recognized by the cytosolic NOD2 [22]. NOD1 and NOD2 are part of the NOD-like receptor (NLR) family that initiates immune responses. Despite variations in the pattern recognition receptor’s location in the cell, ligand recognition, and signaling mechanisms that contribute to the diversity of immune responses, NOD1, NOD2, and TLR4 can induce similar responses in AT, including lipolysis, inflammation, and the onset of IR [23].

Like rodents and humans, dairy cows show a more robust inflammatory response to LPS than LTA. This sensitivity stems from the high reactivity of bovine epithelial cells in the rumen and lactating mammary gland to LPS [24, 25]. When epithelial barriers are compromised, endotoxin levels can rapidly rise, particularly during common acute inflammatory diseases in postpartum dairy cows, such as mastitis and endometritis [26–28]. Disorders that impact the ruminal and intestinal epithelial barrier, such as subacute ruminal acidosis (SARA), can also lead to endotoxemia [29].

Metabolic diseases are also associated with endotoxemic events. For example, we reported that 72.2% of cows diagnosed with clinical ketoacidosis presented high endotoxin content in plasma (0.21 ± 0.03 EU/mL), whereas only 33.3% of healthy postpartum dairy cows had barely detectable blood levels of endotoxin (0.051 ± 0.01 EU/mL) with no signs of disease [30]. Abuajamieh and collaborators reported that dairy cows with endotoxemia one week before parturition developed ketoacidosis during the first week after calving [31]. Cows with ketoacidosis showed a twofold increase in LPS in the blood, accompanied by systemic inflammation, as characterized by high levels of serum amyloid A, LPS binding protein (LBP), and haptoglobin [31]. These findings suggest that endotoxemia may contribute to the pathogenesis of inflammatory and metabolic diseases in dairy cows. Supporting this role, experimentally induced endotoxemia two weeks before and one week after parturition resulted in a higher incidence of displaced abomasum [32]. In a different study, experimentally induced endotoxemia was linked to lactic acidosis in Holstein and Angus pregnant cows [33]. Additionally, fatty liver in cattle could lead to endotoxemia due to the diminished capacity for endotoxin clearance in the liver [34]. For a detailed synopsis of the relationship between endotoxemia and periparturient metabolic disorders in dairy cows, the reader is directed to a comprehensive review by Eckel and Ametaj [15].

## Sources of endotoxin

The primary cause of endotoxemia in dairy cows is endotoxin translocation following increased intestinal permeability [35]. Translocation stems from changes in host-microbiome interactions, allowing the passage of endotoxins normally present within the gastrointestinal tract as part of the gut microbiota [36]. To prevent translocation, mammals have evolved mechanisms to maintain gut barrier integrity that include the secretion of intestinal alkaline phosphatase, antimicrobial peptides, a protective mucus layer, and a complex mucosal immune system. This system involves tolerance for beneficial bacteria and surveillance against pathogens [37, 38]. Intestinal alkaline phosphatase is expressed only in the jejunum of dairy cows, and it dephosphorylates lipid A within the gut, making LPS less active and thus reducing the host’s inflammatory responses [39, 40]. The mucosa’s mucus layer is the first physical barrier encountered by bacteria along the gastrointestinal tract of cows and consists of water (~ 95%) and glycoproteins (1%–10%) [41, 42]. The mucosal barrier also contains antimicrobial peptides and IgA that prevent microbial invasion [43]. Finally, the highly specialized immune cells within the mucosa protect the body from harmful pathogens while maintaining tolerance to beneficial microbes. However, disturbances to these complex mechanisms may result in enhanced gut permeability to endotoxins.

Translocation rates can be affected by alterations in the microbiome composition. In fact, changes in bacterial populations are linked with a rise in endotoxin concentrations in the bloodstream across distinct species [44, 45]. Research in germ-free mice showed the significant role of the gut microbiome in regulating AT and endotoxin translocation. Germ-free mice remain lean on a high-fat and sugar-rich diet, but colonization with bacteria from the cecum of wild-type mice induces obesity [46, 47]. A recent study demonstrated that AT function is linked to microbiome composition in periparturient dairy cows. Therein, Gu and colleagues reported alterations in gut microbiota in postpartum cows with excessive lipolysis (plasma NEFA > 750 µmol/L). These researchers observed changes in at least 14 species of bacteria, and among those, *Lachnospiraceae bacterium, Paraprevotella xylaniphila*, and *Clostridiales bacterium *Marseille-P2846 were the main drivers of microbiome alterations [48]. These changes coincided with a higher abundance of plasma secondary bile acids, synthesized by intestinal bacteria, which was correlated with heightened AT lipolysis. Although the implications of these findings to dairy cows health remain unclear, the authors underscore the association between the microbiome and AT function in cows, as observed in humans and mice [44, 45]. During the periparturient period, ruminal and intestinal microbiota are highly susceptible to changes in their composition, leading to the overgrowth of opportunistic bacteria such as *E. coli* and *Bifidobacterium *sp. This susceptibility is associated with factors such as ruminal acidosis [49], the duration of feeding grain-rich diets [50], heat stress [51], and mastitis [52].

Ruminal and intestinal pH reduction directly triggers changes in cows’ gut bacterial populations. Low GI tract pH is a common characteristic in subacute ruminal acidosis (SARA), a disease associated with alterations in the microbiome composition and intestinal epithelium disturbances, resulting in endotoxin translocation into the bloodstream [49]. SARA induction in late lactation dairy cows led to an increased abundance of *Stenotrophomonas *sp. and *Bacteroides *sp. in the rumen, along with elevated LPS levels in the rumen fluid and the bloodstream [29].

However, pH alone is not the determinant factor in LPS translocation. In vitro studies using ruminal and colon tissues in the presence of LPS demonstrated that when tissue culture pH was reduced to 4.5–5.5, LPS translocated through rumen and colon epithelia independently of pH; however, the presence of LPS favored the translocation of (3)H-mannitol, an intestinal permeability probe [53]. This observation indicates that LPS abundance in the tissue environment may also be a factor in its translocation rate. SARA increases LPS abundance because ruminal acidosis enriches Gram-positive bacteria populations while reducing Gram-negative bacteria. The accumulation of residues from the lysis of Gram-negative bacteria, such as LPS from *Escherichia coli*, in the ruminal fluid disrupts tight junctions and induces cell apoptosis by caspase-3 activation. This process leads to increased permeability to LPS in a dose and time-dependent manner [54]. In mid-lactation cows, SARA increases ruminal endotoxin content from 24,547 EU/mL to 128,825 EU/mL [55] and in peak lactation animals from 28,184 to 107,152 EU/mL [56]. Cows with SARA also present an increase in plasma endotoxin from < 0.05 to 0.52 EU/mL [56]. For a detailed synopsis of the changes in bacterial population and abundance of LPS during SARA, the reader is directed to a comprehensive review by Monteiro and Faciola [57].

Similar to SARA, heat stress is associated with endotoxemia. The mechanism leading to bacterial translocation during heat stress is altered intestinal permeability. This explains why animals experiencing heat stress exhibit several overlapping metabolic and physiological changes analogous to those observed in endotoxemia [58]. Endotoxemia occurs during heat stress and results from structural alterations in the gastrointestinal system. The blood flow to the visceral organs is drastically reduced, resulting in hypoxia and enterocyte death and consequently increasing intestinal permeability [59]. These intestinal alterations and the recruitment of different immune cells in the intestine were described in lactating cows with heat stress, leading to endotoxemia [60, 61]. Both conditions can alter various physiological parameters, including changes in body temperature, metabolic rate, and inflammatory responses [58].

In dairy cows, common postpartum diseases of bacterial origin, such as metritis, mastitis, and pneumonia, are also sources of circulating endotoxin [62]. Uterine inflammation is associated with high circulating endotoxin levels during the periparturient period. In cases of naturally occurring endometritis, endotoxin is present in the plasma in a severity-dependent manner. In one study, 17% of cows diagnosed with mild endometritis (mucopurulent lochia) were found to have endotoxemia, whereas 100% of cows diagnosed with severe endometritis (sanguine-purulent lochia) exhibited endotoxemia [26]. Intrauterine infusion of 5 µg/kg of BW of *E. coli* in cows resulted in the detection of endotoxin in plasma, whereas saline-infused controls consistently tested negative for LPS [63]. Cows with naturally occurring mastitis exhibited 18-fold greater plasma endotoxin levels than healthy controls [28]. Similarly, experimentally induced mastitis increases plasma endotoxin levels 10-fold [64]. While experimental models of endotoxemia can contribute to understanding its pathophysiology, it is crucial to distinguish between experimentally induced and naturally occurring endotoxemia to comprehend the complexity of the host response.

The LPS used in experimental endotoxemia in dairy cows is derived from *E. coli*, while natural LPS originates from various Gram-negative bacterial populations [57]. The extensive diversity in the chemical composition of the polysaccharide region (O-antigen) and lipid A, gives rise to a wide array of natural structural variants, leading to varying immune responses and degrees of pathogenesis [65]. The intricate nature of the endotoxemia response in cows is influenced by factors such as dose, LPS strain, and individual variability, highlighting that not all cows exhibit identical responses to endotoxin [66]. Recent research showed that bovine ruminal epithelial cells exhibit differential responses to experimental LPS from *E. coli* compared to ruminal LPS [67]. During a continuous exposure of 6 h, the inflammatory response of ruminal epithelial cells was more pronounced in reaction to experimental LPS than ruminal LPS. However, ruminal epithelial cells develop tolerance to sustained ruminal LPS but not to synthetic endotoxin. These findings highlight the intricacies of the various responses during endotoxemia and emphasize the limited understanding of the mechanisms involved in host-bacteria interaction.

## Endotoxin translocation mechanisms

Endotoxins in dairy cattle can surpass various defense mechanisms linked to the epithelial barrier, resulting in enhanced translocation from the gastrointestinal tract, mammary gland, and uterus into the blood. However, there are additional routes by which endotoxins may enter the bloodstream in smaller amounts, including through the respiratory tract and skin lesions [68]. While limited research exists on the molecular mechanisms of endotoxin translocation in ruminants, recent studies suggest that these mechanisms are likely similar between rodents and bovines [27, 69, 70].

**Fig. 1:**
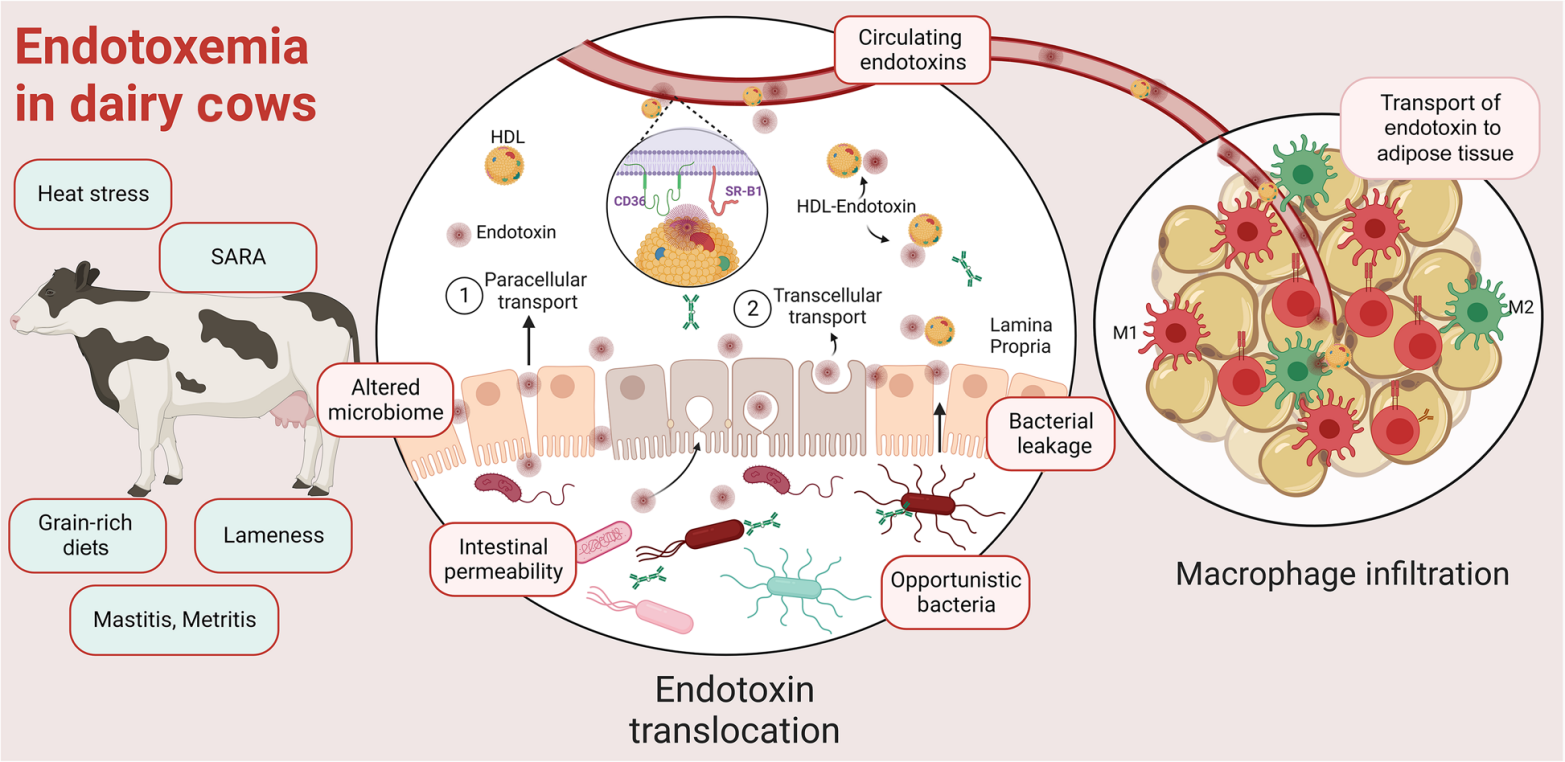
Endotoxin sources and transport in dairy cows. Dairy cows with conditions such as heat stress, subacute ruminal acidosis (SARA), lameness, mastitis, metritis, or consuming grain-rich diets are more likely to have circulating endotoxins in their bloodstream, a condition called endotoxemia. Elevated blood endotoxin levels correlate with alterations in microbiome composition, the proliferation of opportunistic bacteria, heightened intestinal permeability, and bacterial leakage. Endotoxins enter the circulation through (1) paracellular and (2) transcellular transport, facilitated by their interaction with high-density lipoproteins (HDL). HDL-endotoxin complexes enter blood circulation by scavenger receptor of the class B type I (SR-BI) or fatty acid translocase CD36. HDL-endotoxin complexes reach the adipose tissue, where endotoxins induce the polarization of macrophages towards the M1 phenotype while decreasing the abundance of M2, thus promoting inflammation. Created with BioRender.com

The first mechanism is passive diffusion by increased paracellular permeability, a process commonly referred to as “leaky gut” (Fig. 1). The paracellular transport of endotoxins is triggered by the alteration of tight junctions, comprised of claudins and occludin, which regulate paracellular permeability [71]. In late lactation dairy cows, a rich-grain diet led to increased intestinal content of LPS and reduced expression of claudin-4 and ZO-1 in the ileal epithelium, indicating damage to tight junctions and increased intestinal permeability [72]. Similarly, in goats, induction of SARA led to increased ruminal LPS, compromising ruminal barrier integrity. This impairment subsequently reduced the expression of claudin and occludin in the rumen, facilitating LPS translocation [69]. Also, altering tight junctions in mammary epithelial cells leads to an impaired blood-milk barrier. The disruption is likely attributed to the decreased transcription and translation of claudins in bovine mammary cells in the presence of both LPS and LTA [27]. Therefore, endotoxins may enter through the paracellular pathway in the presence of mucosal injury [73]. The molecular pathways involved in paracellular and tight junction dysfunction were recently reviewed by Horowitz et al. [74].

The second mechanism involves the active transport of endotoxin through the transcellular pathway [75]. Both in vivo and in vitro studies employing electron microscopy demonstrate that endotoxin is taken up by jejunal and colonic enterocytes and crosses the basal epithelium via endocytic pathways [76, 77] (Fig. 1). TLR4 facilitates endotoxin internalization by enterocytes in an MD-2-dependent manner [78]. The process is TLR4-mediated, as evidenced by the absence of LPS internalization in TLR4^-/-^ mice [79]. Furthermore, in mice, endotoxin may enter the enterocyte through lipid transport mechanisms involving the scavenger receptor class B type I (SR-BI) and fatty acid translocase CD36 [79, 80].

## Endotoxin trafficking

Once LPS enters the enterocyte, its trafficking throughout the intestinal epithelial layer depends on different intracellular organelles before being exported into the circulation by transcytosis. Transcytosis is the vesicular transport of substances from the apical membrane to the basal membrane involving the uptake (endocytosis), intracellular transport and release (exocytosis). Inside the enterocytes, endotoxins are transferred to the endoplasmic reticulum and then transported to the Golgi apparatus to be incorporated into chylomicrons [81]. These large lipoproteins are crucial in transporting endotoxin into the lymphatic system [79]. This clarifies why, in some cases, healthy individuals may briefly experience endotoxemia after consuming high-fat meals, as lipids are highly effective carriers of endotoxin [82]. Once LPS has been incorporated into lipoproteins and enters the blood stream, it reaches the liver. In this organ, endotoxin triggers the production of acute-phase proteins [83]. In dairy cows, the concentrations of acute-phase proteins in the bloodstream during endotoxemia exhibit an LPS dose-dependent relationship [66].

In the bloodstream, approximately 60% of endotoxins bind to HDL. This complex has an affinity for the liver, where LPS can be processed for elimination. The remaining fraction is associated with LPS binding protein (LBP) or soluble CD14 [84]. When bound to HDL, endotoxin’s capacity to stimulate the immune system significantly decreases, leading to a notable reduction in cytokine production in animals with high levels of this lipoprotein [85]. This potential benefit of HDL has been observed in both human and rodent models, where HDL was shown to mitigate inflammation during endotoxin challenges [86, 87]. HDL accounts for up to 75.7% of the lipoproteins in dairy cows, and the ratio HDL:LDL ranges 6.8–7.9 [88]. In contrast, humans have a lower abundance of HDL (25%–33%) and a reduced HDL:LDL ratio of 0.3 to 0.4, making them more susceptible to endotoxemic events [89]. This distinctive feature may contribute to cattle’s resistance to endotoxemia compared to other species.

The reduced immunostimulatory effect of the HDL-endotoxin complex can be attributed to structural changes in endotoxin itself, causing specific epitopes to become less exposed or hidden entirely from immune cells upon HDL binding [85]. However, HDL undergoes continuous remodeling as it exchanges lipids and apolipoproteins with other lipoproteins and tissues. HDL-endotoxin complexes are unstable, and endotoxin is often transferred from HDL to LDL by the phospholipid transfer protein and LBP. Moving endotoxin from HDL to LDL is a process that works in a time- and dose-dependent manner and results in the remodeling of HDL [90]. This is thought to be a contributing factor to the plasma lipoprotein dyslipidemia observed during the acute phase response to endotoxemia. Additionally, the formation of LBP-endotoxin complexes serves a dual purpose, provoking an immune response and tempering excessive inflammation [91]. When LBP is low in quantity, the interaction between endotoxin and immune cells intensifies, provoking a systemic immune response. Conversely, higher LBP concentrations are responsible for inhibiting LPS-induced cellular stimulation during the acute phase of infection [91].

The transfer of LDL- and HDL-endotoxin complexes from the bloodstream to endothelial cells is facilitated by the SR-BI and CD36 receptors [92]. Importantly, caveolin and cavin family proteins, known to be involved in various physiological functions such as cell signaling and endocytosis, are colocalized with SR-BI and CD36 in the cell membrane [93]. Remarkably, caveolae constitute up to 50% of the plasma membrane surface in both adipocytes and endothelial cells [94]. These data suggests that the binding of HDL-endotoxin to SR-BI or CD36 may lead to an elevated transportation of endotoxin into the adipocytes. Although AT is considered a site of toxins accumulation, the dynamics of endotoxin buildup in ruminants’ AT warrant further investigation [95].

## Effects of endotoxemia on AT

### Inflammation

Endotoxemia results in local and systemic inflammation that is associated with marked alterations in the metabolism of lipids, proteins, and carbohydrates [45]. The augmented production of inflammatory mediators following endotoxin exposure may induce a cascade of altered functionalities in multiple organ systems, leading to organ failure or, in severe cases, death. Among the mediators produced, cytokines are crucial in regulating host responses to endotoxemia [96, 97]. Some cytokines directly affect AT metabolism, while others indirectly enhance the release of metabolically active hormones — catecholamines, cortisol, glucagon, and adrenocorticotropin — that influence AT function [45].

**Fig. 2:**
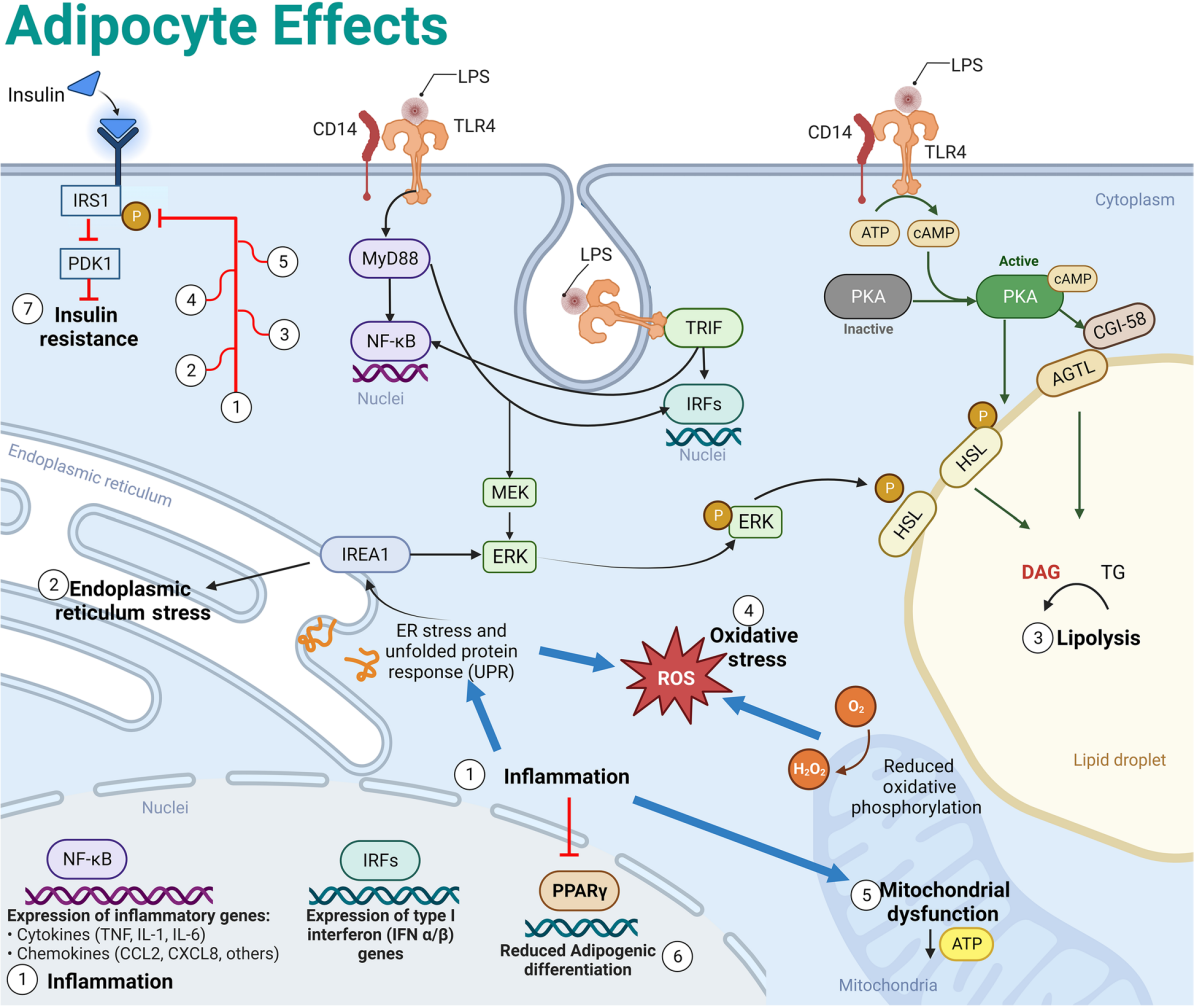
Summary of endotoxin effects on adipocytes. Lipopolysaccharide (LPS) is recognized by CD14 molecules, and its effects depend on TLR4 signaling. The activation of MYD88 and TRIF pathways result in enhanced expression of transcription factors (nuclei) including NF-κB and IRF. NF-κB enhances the expression of pro-inflammatory cytokines leading to inflammation (1). Inflammation induces endoplasmic reticulum (ER) stress (2), leading to the activation of unfolded protein response (UPR). Products from the UPR such as IREA1 are necessary for ERK1-mediated activation of hormone-sensitive lipase (HSL). Lipolysis (3) is also activated by TLR4 signaling by enhancing PKA and the subsequent activation of HSL and adipose triglyceride lipase (ATGL). Inflammation and UPR contribute to elevated oxidative stress (4) by higher production of reactive oxygen species (ROS). Inflammation is the main contributor of mitochondrial dysfunction (5), leading to lower production of ATP. Inflammation inhibits the activity of peroxisome proliferator activated receptor gamma (PPARγ), resulting in lower adipogenic capacity (6). Together, (1) Inflammation, (2) ER stress, (3) Lipolysis, (4) Oxidative stress, and (5) Mitochondrial dysfunction lead to (7) Insulin resistance. Created with BioRender.com

The major inducer of immune activation in response to endotoxin is TLR4 (Fig. 2). Once bound to TLR4, endotoxins trigger AT inflammation via MYD88-dependent or -independent (TRIF-dependent) pathways [98]. MYD88-dependent signaling results in early activation (i.e., early phase) of the transcription factor nuclear factor-kappaB (NF-κB), which leads to the rapid production of inflammatory cytokines. Activation of the TRIF-dependent pathway concludes with a late phase of NF-κB activity, which sustains prolonged activation of NF-κB [99]. This late phase is particularly relevant in chronic inflammation and long-term cellular immune responses. Both MYD88 and TRIF pathways are essential for inflammatory responses in AT, including the polarization of resident macrophages following LPS stimulation [100]. Activating NF-κB is necessary for AT cytokine production since NF-κB inhibition reduces IL-6 and IL-8 expression in adipocytes [96].

In dairy cows, AT are immunologically responsive to endotoxin. For example, challenging AT explants with LPS (20 µg/mL/2 h) resulted in higher transcription of IL-6 and tumor necrosis factor alpha (TNFα), especially in mesenteric depots [101]. Our group demonstrated that LPS (20 µg/mL media) increases transcription of pro-inflammatory cytokines (*CCL2, CD204, IL-6, IL-8, SOCS1*) after 3 h of challenge and reduces the expression of anti-inflammatory mediators (*IL-10, STAT3*) after 7 h of exposure in subcutaneous AT [102]. In other domestic species and humans, endotoxemia induces AT inflammation through the exact mechanisms [16, 103, 104]. The activation of AT inflammatory responses is not exclusive to LPS. PGN activates the NOD1 ligand in AT cells, including adipocytes, resulting in an increased pro-inflammatory profile (*IL-1β, IL-18, IL-6, TNF*) mediated by PKCδ, IRAK1/4, and NF-κB activation [23, 105]. The inflammatory responses within the AT may vary due to the diverse types of cells present in the organ. For instance, adipocytes are less likely than macrophages to elicit a strong inflammatory response to LPS, primarily because macrophages have a higher density of TLR receptors [106].

### AT macrophage infiltration

Macrophages are the predominant immune cells within the AT, and regulate the organ’s immune response, energy metabolism, and mitochondrial function. AT macrophages (ATM) originate from both circulating monocytes and resident macrophages, and diverse mechanisms, including endotoxin, mediate their activation. ATM are broadly classified as M1 (classically activated) with pro-inflammatory characteristics or M2 (alternatively activated), which exhibit anti-inflammatory properties (macrophage phenotypes and subtypes are reviewed comprehensively by Caslin et al. [107]). Endotoxin polarizes macrophages toward the M1 phenotype through TLR4-dependent activation [108]. M1 polarization results in enhanced secretion of pro-inflammatory cytokines such as IL-1β, TNF, IL-12, IL-18, and IL-23. During low-grade endotoxemia and intense NEB, dairy cows have a greater abundance of M1 in AT [30]. ATM infiltration is present in transient NEB states without endotoxemia, but the tendency for greater M1 polarization is not observed [11].

During metabolic and inflammatory diseases, bovine ATM exhibit M1 polarization and aggregate forming “crown-like structures” (CLS) [30]. CLS are accumulations of ATM that localize dead adipocytes and their remnants to limit the lipotoxic effects of TG and other neutral lipids abundant in adipose cells [109]. M1 polarization of ATM is common in cows with displaced abomasum and clinical ketosis where ATM infiltrate and aggregate in omental and subcutaneous AT [14, 30]. Similarly, in humans and rodents with high adiposity and IR, the infiltration of M1 ATM is frequently observed [110]. Typically, the proportion of M2 and M1 ATM is used as an indicator of AT inflammation and functionality. In healthy AT, M2 macrophages outnumber M1 macrophages by a ratio of 4:1 [111]. However, during inflammatory responses the proportion of M1 is enhanced, resulting in a 1.2:1 ratio [111]. A key mechanism that controls macrophage polarization was recently discovered. The glucose-regulated protein 94 (GRP94), an endoplasmic reticulum chaperone, is a novel regulator of M1 macrophages in murine AT [112]. The specific deletion of this receptor in mice results in a small number of M1 and enhanced insulin sensitivity, however, more research is needed to understand the complete mechanisms driving M1 polarization in AT during endotoxemia and associated diseases in dairy cows.

### Endoplasmic reticulum stress

Upon exposure to endotoxins, cells undergo a cascade of responses that extend into the organelles, impacting critical processes within the endoplasmic reticulum (ER). The ER is essential for lipid and protein synthesis, calcium (Ca^2+^) storage, protein folding, and other post-transcriptional changes [113]. Additionally, the ER participates in inflammation, insulin signaling, cell death, and proliferation. Disruption of cellular equilibrium by endotoxemia, inflammation, and oxidative stress leads to the development of ER stress, which, in turn, activates the unfolded protein response (UPR) signaling pathway [114]. The elements of the UPR/ER stress response include inositol-requiring protein 1 (IRE1), activating transcription factor 6, and protein kinase RNA-like ER kinase (Fig. 2). Although UPR is considered a compensatory mechanism to re-establish cellular homeostasis, continuous ER stress may result in cell death. In bovine mammary epithelial cells, LPS activates ER stress by increasing the expression of glucose-regulated protein 78 kDa (GRP78) and activating transcription factor 6 (ATF6). A similar phenomenon is observed in humans and rodents, where the activation of TLR4 by endotoxin in adipocytes leads to ER stress and inflammation [113, 115]. While ER stress during endotoxemia in bovine AT remains unexplored, ketotic cows exhibit ER stress within their AT. This process was evident through increased protein expression of IREA1 and ATF6, which correlates with enhanced AT inflammation [116]. The activation of IRE1 leads to upregulation of c-Jun N-terminal kinase (JNK) and NF-κB, which induce the transcription of inflammatory genes, increase oxidative stress, and caspase-dependent apoptosis. ER stress impairs adipocyte metabolic function, leading to IR and reduced synthesis and secretion of adiponectin [114, 117]. Adiponectin is exclusively produced by adipocytes, and it enhances insulin sensitivity and has anti-inflammatory properties. In dairy cows, lower secretion of adiponectin is directly correlated with diminished insulin sensitivity [118]. Bovine adipocytes treated with TNF reduced their adiponectin production. This outcome was attributed to the diminished expression of ER protein 44 (ERP44), ER oxidoreductase 1α (ERO1A), and peroxisome proliferator activated receptor gamma (PPARγ) [119]. However, considering the impact of endotoxins on ER stress, and TNFα activation, it is reasonable to propose that inflammation triggered by endotoxemia may be a contributing factor to reduced adiponectin levels following the activation of ER stress pathways.

## Mitochondrial dysfunction and oxidative stress

Beyond their role as adipocyte’s powerhouse, mitochondria regulate various cellular events, including lipogenesis, apoptosis, and innate immune responses [120]. For example in late lactation cows, increased lipogenesis in adipocytes is positively associated with higher mitochondrial DNA (mtDNA) copies and biogenesis and with higher body condition scores [121]. This link is reinforced by mitochondria’s role in adiponectin synthesis, an adipokine with antilipolytic properties [122]. In mice, endotoxins disrupt the equilibrium of mitochondria, affecting processes such as ATP production through the electron transport chain and the tricarboxylic acid cycle [123]. In humans, a recent study showed that gut-derived endotoxemia reduced mitochondria biogenesis by 81.2% within the AT, resulting in adipocyte dysfunction [124]. Therefore, it is a plausible hypothesis that endotoxemia in dairy cows may induce similar mitochondrial alterations.

Mitochondrial dysfunction during endotoxemia impairs adipocyte metabolism and induces a cascade of detrimental effects including oxidative stress, cell death, inflammation, and cellular dysfunction [125]. Inflammation derived from endotoxins stimulates adipocyte receptors, such as TLR4, NOD1, and NOD2, triggering stress signals that diminish oxidative phosphorylation and electron transport chain activity. This disruption leads to decreased ATP production, increased anaerobic oxidation, and higher levels of glycolysis and lactate [126]. Similar alterations in cellular oxygen supply are observed in humans with obesity [127] and over-conditioned dairy cows, resulting in reduced blood flow, low oxygen availability, altered mitochondrial DNA copies, and increased transcription of angiogenic factors, leading to inflammation and cell death in adipose tissue of non-lactating dairy cows [128].

The balance between reactive oxygen species (ROS) production and antioxidant defenses activity maintains cellular homeostasis. ROS are necessary for redox signaling. For example, ROS activate innate immune responses and turn on PPARγ-mediated lipogenic and adipogenic responses [129]. Antioxidants limit these responses. However, exposure to endotoxins induces excessive ROS production, disrupts redox balance, and leads to protein, lipid, nuclear DNA, and mitochondrial DNA damage within adipocytes [130]. For instance, in adipocytes exposed to endotoxin, the activity of inducible nitric oxide synthases (iNOS) is enhanced, leading to higher production of nitric oxide (NO) [131]. In mice, elevated levels of NO result in impaired AT function by enhanced cellular damage and apoptosis due to alterations in DNA and protein structure, and the disruption of the electron transport chain [132]. In postpartum dairy cows, lipolysis triggered by endotoxemia leads to the production of pro-inflammatory oxylipids, creating a pro-oxidative environment in AT that may increase disease risk [133].

## Endotoxemia alters lipid metabolism

### Lipolysis

In AT, the breakdown of the ester bonds between fatty acids and their glycerol backbone is performed by 3 specific lipases: (1) adipose triglyceride lipase (ATGL), which hydrolyzes TG to generate NEFA and diacylglycerol [134]; (2) Hormone-sensitive lipase (HSL), which is a more versatile enzyme with the capacity to hydrolyze TG, DG, and monoacylglycerol, breaking them down into mono and diglycerides, fatty acids, and glycerol [135]; and (3) Monoglyceride lipase, which hydrolyzes monoacylglycerol into NEFA and glycerol [136]. During fed states, there is a continuous rate of lipolysis that releases fatty acids for cellular functions, alongside continuous re-esterification, known as basal lipolysis. The primary regulator of basal lipolysis is ATGL, whose expression is conditioned by adipocyte size and TG content in human, bovine, and rodent AT [137, 138]. However, additional factors such as sex, age, physical activity, and fat location have been shown to influence lipolytic activity [139]. During periods of NEB, HSL is the main lipase contributing to stimulated lipolysis [140]. After activation by catecholamines, β-adrenergic receptors stimulate the production of cAMP, subsequently leading to the activation of protein kinase A (PKA). PKA then phosphorylates perilipin and HSL, resulting in TG breakdown [141].

Guinea pigs were among the first animals in which the lipolytic effect of endotoxins was observed. Pond and Mattacks [142] found that AT surrounding lymph nodes exhibited enhanced lipolysis in the presence of lymphoid cells stimulated with 50 µg/mL of LPS. The same study revealed that AT proximal to lymphatic nodes presented higher lipolysis than fat depots collected in other areas, suggesting a systemic response. These findings were subsequently confirmed in vivo, as spontaneous lipolysis was observed in adipocytes around the popliteal lymph node of guinea pigs after a subcutaneous LPS injection (1 µg/100 g body mass) [143]. This study evidenced a peak in lipolysis between 6 and 9 h after the LPS challenge, which gradually declined over 24 h.

Similarly in cattle, LPS induced lipolysis in AT from both lactating and non-lactating cows [102]. In periparturient dairy cows, LPS (20 µg/mL) increased lipolysis by 67% ± 12% compared to basal levels. The same dose resulted in 115% ± 18% more lipolysis, compared to basal conditions, in AT from non-lactating dairy cows [102]. The lipolytic response to endotoxin in dairy cows may be orchestrated through the activation of pro-inflammatory cytokines. Notably, TNF activates lipolysis in bovine adipocytes by initiating NF-κB and JNK signaling pathways [144].

Bovine, murine, and human adipocytes respond to the lipolytic effect of endotoxins in doses ranging from 0.01 to 10 µg/mL [96, 145, 146]. Endotoxin-induced lipolysis is dependent on TLR4 activation in dairy cows’ adipocytes. Experiments conducted in bovine adipocytes lacking TLR4 demonstrated that LPS increase lipolysis by 72.6% ± 16% only in cells expressing the receptor [146]. The same observations were reported in TLR4^−/−^ mice, where endotoxin induced lipolysis, exclusively in wild-type animals [96]. In dairy cows, the transcriptomic profile of AT revealed a link between TLR4 activation and lipolysis in the early postpartum period, evidencing—for the first time in vivo—a relationship between endotoxins and enhanced lipid mobilization [147]. Importantly, lipolytic activity induced by endotoxins is triggered by two intracellular lipolytic pathways: canonical and inflammatory lipolysis pathways.

### Canonical pathway

The canonical pathway of lipolysis involves cellular responses to hormonal stimulation (e.g., adrenergic), leading to the activation of PKA and consequently HSL. In the 1970s, Hikawyj-Yevich and colleagues demonstrated the activation of the canonical pathway following exposure to endotoxins [148]. Their research showed endotoxin (0.16 µg/mL) increased the levels of cAMP amplifying hormonal-stimulated lipolysis in primates. In a separate study, human adipocytes were pre-treated with inhibitors targeting PKA (H-89) or HSL (CAY10499). Subsequently, when these pre-treated cells were exposed to LPS (100 ng/mL, 24 h), lipolysis was effectively inhibited [145]. Similarly, PGN (10 µg/mL) activated canonical lipolysis in murine AT, relying on NOD1 ligand activation and PKA/HSL dependent mechanisms [149]. HSL is phosphorylated by PKA at multiple serine residues including Ser-659, Ser-660, Ser 563, Ser 649, Ser-650, and Ser-552 [150]. Although LPS phosphorylates Ser650 and tends to phosphorylate Ser552 in humans [151], the effect of endotoxin on the canonical lipolytic pathway is not defined in dairy cows.

### Inflammatory pathway

The lipolytic inflammatory pathway is triggered by inflammatory molecules. In murine adipocytes, 1 µg/mL of LPS resulted in higher phosphorylation of the Raf-1 signaling, an important kinase in the mitogen activated protein kinase (MAPK) pathway. In addition, LPS induced the phosphorylation of MEK1/2 (ERK1/2 MAPK), which results in rapid activation of HSL and lipolysis [96]. To demonstrate the dependency of this pathway on lipolysis, the inhibition of MEK-ERK1/2 with either PD98059 or U0126, resulted in inhibition of glycerol release following LPS stimulation. Similar findings were reported in adipocytes stimulated with PGN (10 µg/mL), where ERK activation is partially involved in lipolysis [149]. ERK phosphorylates HSL Ser-600 and Ser-589 in humans and rodents [150], in line with this, our group showed that AT of dairy cows exposed to LPS during 7 h, increased the phosphorylation of ERK1/2 parallelly with lipolysis activation and HLS phosphorylation at Ser-563, which may uncover differences in critical residues of HSL among species [102]. New evidence has demonstrated that ERK1/2 promoted serine phosphorylation of the beta3 adrenoreceptor, which results in enhanced lipolysis [152].

The exact mechanisms of ERK1/2 mediated lipolysis appear to be related to ER stress. Pharmacological induction of ER stress in adipocytes triggers lipolysis through the activation of cAMP/PKA and ERK1/2 signaling pathways and concomitant higher perilipin phosphorylation [153]. However, the distinction between lipolysis induced by immune responses and hormone/adrenergic signals was just recently uncovered. It was unclear if inflammation uses a specific kinase or shares hormonal pathways to activate lipolysis mediated by ERK1/2/PKA/Lipases. Foley and colleagues analyzed different inflammatory effectors such as LPS, PGN, and TNF-α, demonstrating that inflammatory agents-induced lipolysis is mediated by ER stress [154]. The activation of IRE-1 is indispensable for inflammatory but not for canonical lipolysis (Fig. 2).

The NF-κB inflammatory pathway is also an inducer of lipolysis. Treating adipocytes with 100 ng/mL of LPS for 24 h triggered lipase activation and FFA release in humans. Pre-treating adipocytes with inhibitors of NF-κB pathway components IKKβ and NF-κB abolished LPS-induced lipolysis [145]. At least three mechanisms behind NF-κB activation of lipolysis relate to the downstream activation of TNF-α. First, TNF-α downregulates the activity of phosphodiesterase 3B, an enzyme that reduces cAMP [155]. Second, TNF-α induces perilipin phosphorylation in human and mouse adipocytes, leading to the de-coating of lipid droplets; this in turn, increases the access of lipases to TG for hydrolysis [96, 156]. A third mechanism involves the downregulation of GTP binding protein Gα by TNF-α, which increases cAMP concentrations [157].

Prostaglandins synthesized by cyclooxygenases (COX) also activate lipolysis. Endotoxins are potent modulators of COX-2 in AT [158]. COX-2 is the rate-limiting enzyme for the biosynthesis of prostaglandins (PGs), including PGD2, PGE2, PGF2a, and prostacyclin (PGI2) [159]. Treating 3T3-L1 adipocytes with PGE2 induces lipolysis [160]. The PGE2 receptor EP4 mediates this lipolytic signal by phosphorylating PKA and HSL downstream, resulting in TG hydrolysis [161]. The COX-2/PG axis plays a critical role in regulating AT inflammation and lipid metabolism. For instance, COX2 inhibition results in enhanced fat deposition in rodents. In fact, indomethacin, a COX inhibitor, is commonly used to stimulate adipogenesis in mesenchymal stem cells [162]. However, whether endotoxin-induced COX-2 in adipocytes is implicated in lipid mobilization in dairy cows is unclear.

Reports in dairy cows indicate that intravenous LPS infusion activates lipolysis, causing increased plasma NEFA levels. Waldron et al. [163] noted a peak in NEFA levels 2 h after infusing 1 µg/kg of LPS in mid-lactation cows (150–220 days in milk). Similarly, in non-pregnant lactating cows (169 ± 20 DIM), circulating NEFA increased during continuous LPS (up to 0.148 µg/kg) infusion for 8 d [164]. The lipolytic response to LPS in vivo depends on continuous endotoxin stimulation. Periparturient dairy cows receiving intermittent infusion of LPS before parturition (−14 d and −10 d) and after calving (d 3 and d 7) did not present changes in plasma NEFA. Differences in the lipolytic response to endotoxin could be attributed to the LPS type and the metabolic status of the animals. However, the mechanisms behind this response remains to be investigated.

### Lipid accumulation

Endotoxin-induced inflammation affects adipogenesis and lipogenesis in adipocytes. Adipogenesis is when committed mesenchymal stem cells or preadipocytes differentiate into mature adipocytes. Adipogenesis increases mammal AT lipid storage capacity [165]. The literature presents conflicting evidence about the adipogenic potential of AT during endotoxemia. In vitro studies suggest LPS (0.1 up to1 µg/mL) reduces adipogenesis in primary stromal vascular fraction cells isolated from murine inguinal AT [166, 167]. One mechanism for reduced adipogenesis is the inhibition of AMPK phosphorylation, which suppresses the expression of adipogenic factors, including PPARγ [17, 167]. Conversely, 3T3-L1 cells treated with 20 µg/mL of endotoxin increased cell proliferation and adipogenesis via JAK2/STAT and AMPK [168]. A 4-week in vivo study induced metabolic endotoxemia in mice by LPS infusion. This led to increased proliferation of adipocyte progenitors, although their adipogenic potential remained limited [17].

In contrast to adipogenesis, lipogenesis is the formation of new FA and TG molecules within a mature adipocyte. Lipogenesis results in hypertrophy of adipose cells [165, 169]. The impact of endotoxemia on lipid accumulation varies based on exposure duration and intensity. In obese rodents and humans, elevated endotoxin levels in blood are associated with reduced expression of lipid accumulation markers, including PPARγ, SCD, SREBP1, FABP4, FASN, and LEP in AT [170]. Mice fed a high-energy diet for eight weeks following a 4-week LPS challenge had increased body weight, higher AT mRNA expression of *TNF-α*, and glucose intolerance compared to saline-infused mice [17]. Remarkably, the weight increase was due to AT expansion rather than a size increase in other organs. The above findings highlight the long-term effects of endotoxemia on AT function. Initially, adipocyte hyperplasia was enhanced, and subsequently, hypertrophy developed due to the increased energy availability. There is a lack of research about the effects of endotoxemia on AT adipogenic and lipogenic potential in dairy cows.

### Insulin resistance

IR in AT occurs when physiological concentrations of insulin have reduced biological responses, resulting in decreased glucose uptake, diminished lipogenesis, and failure to inhibit lipolysis [171]. In dairy cows, transient multiorgan IR is one of the major adaptations occurring during the periparturient period that ensures sufficient supply of glucose for the uterus and mammary gland [172]. However, it is important to note that insulin resistance may also arise as part of pathological processes, increasing the risk for metabolic diseases [173, 174]. Pathologic IR can be induced by inflammation, particularly through TLR4 activation by endotoxins. For details on IR’s molecular biology, readers are referred to specific reviews on this topic [175, 176].

Insulin signaling involves two main cellular pathways: (1) The phosphatidylinositol 3-kinase (PI3K)-Akt pathway that mediates insulin’s effects on glucose uptake and metabolic functions; (2) The Ras-MAPK pathway, a transcriptional regulator that interacts with the PI3K-Akt pathway to control cell growth and differentiation (reviewed in [177]). Insulin receptor substrate 1 (IRS1) serves as a common intermediate in both pathways. IRS1 phosphorylation at serine 307 can impede downstream signaling [178]. Inflammatory factors like TNFα, JNK, and suppressor of cytokine signaling (SOCS) proteins negatively regulate IRS proteins, promoting their degradation and contributing to IR [179]. Dysfunction of IRS proteins results in the inhibition of protein kinase B (Akt). Akt, a serine/threonine kinase, becomes activated (phosphorylated) as a downstream event following the activation of the insulin receptor [180]. Therefore, Akt phosphorylation intensity reflects insulin activity.

In periparturient dairy cows with endotoxemia and clinical ketosis, AT showed reduced Akt phosphorylation and IR [30]. Similarly, our group demonstrated that AT from non-lactating cows develop IR when exposed to LPS. This impairment can be attributed to reduced phosphorylation of Akt [102]. The primary contributors to IR in monogastric animals are the inflammatory mediators triggered by endotoxins. For example, murine adipocytes exposed to TNFα had higher IRS1 phosphorylation, attenuating insulin signaling activation [181]. Similarly, enhanced JNK activity induced by inflammation leads to IR. JNK1 knockout animals have reduced serine phosphorylation of the IRS1 and thus limited development of IR [182]. Oxidative stress also leads to IR by enhancing iNOS activity resulting in IRS1 degradation [183] (Fig. 2). NOS also reduces Akt signaling by s-nitrosylation of cysteine residues [184]. *CCL2*, also known as monocyte MCP-1, mediates the development of IR in obese mice. When *CCL2* knockout mice are fed high-fat diets, these animals present limited ATM infiltration and improved insulin sensitivity. The mechanisms leading to IR in the AT of dairy cattle are expected to be similar to those in monogastric animals; however, further research is needed to confirm this conjecture.

## Conclusions

In dairy cows, periparturient metabolic stress increases their susceptibility to metabolic and inflammatory diseases. AT responds to metabolic stress by providing FA as energy substrates to offset NEB. Endotoxemia can induce inflammation in AT, disrupting a delicate physiological balance and increasing susceptibility to metabolic stress. Similar to monogastric animals, TLR4 activation by endotoxins in cattle triggers various stress responses, such as increased cytokine production, pro-inflammatory macrophage polarization (M1), ER stress, mitochondrial dysfunction, and oxidative stress. Endotoxin exposure in AT also affects lipid mobilization by enhancing lipolysis and limiting adipogenesis. Endotoxemia-driven disruptions in AT function may result in IR increasing dairy cows’ susceptibility to metabolic disease. Additional research is required to determine the precise mechanisms underlying these effects and to explore potential preventive interventions to mitigate the effects of endotoxemia and bacteremia in dairy cows.

## Abbreviations

AT: Adipose tissue
ATGL: Adipose triglyceride lipase
ATIR: Adipose tissue insulin resistance
ATM: Adipose tissue macrophage
ATP: Adenosine triphosphate
CCL2: Chemokine ligand 2
CD204: Cluster of differentiation 204
COX: Cyclooxygenase
DG: Diacylglycerol
ER: Endoplasmic reticulum
EU: Endotoxin units
GRP94: Glucose-regulated protein 94
HDL: High density lipoproteins
HSL: Hormone sensitive lipase
IL-10: Interleukin-10
IL-18: Interleukin-18
IL-1β: Interleukin-1β
IL-6: Interleukin-6
IL-8: Interleukin-8
iNOS: Inducible nitric oxide synthase
IR: Insulin resistance
IRAK: Interleukin-1 receptor-associated kinase
IRE-1: Inositol-requiring protein 1
IRS1: Insulin receptor substrate 1
JNK: C-Jun N-terminal kinase
LPS: Lipopolysaccharide
MAPK: Mitogen activated protein kinase
MCP-1: Macrophage chemotactic protein 1
meso-DAP: D-glutamyl-meso-diaminopimelic acid
MGL: Monoglyceride lipase
MYD88: Myeloid differentiation primary response 88
NEB: Negative energy balance
NEFA: Non-esterified fatty acids
NF-κB: Nuclear factor-kappa B
NO: Nitric oxide
NOD1: Nucleotide oligomerization domain 1
NOS: Nitric oxide synthases
PG: Prostaglandin
PGN: Peptidoglycan
PI3K: Phosphatidylinositol 3-kinase
PKA: Protein kinase A
PKCδ: Protein kinase C-δ
PPARγ: Proliferator activated receptor gamma
RNS: Reactive nitrogen species
ROS: Reactive oxygen species
SARA: Subacute ruminal acidosis
SOCS: Suppressor of cytokine signaling
SR-BI: Scavenger receptor of the class B type I
STAT3: Signal transducer and activator of transcription 3
TG: Triglycerides
TLR4: Toll-like receptor 4
TNF: Tumor necrosis factor
TRIF: TIR-domain-containing adapter-inducing interferon-β
UPR: Unfolded protein response
